# Real-Time Detection of Distracted Walking Using Smartphone IMU Sensors with Personalized and Emotion-Aware Modeling

**DOI:** 10.3390/s25165047

**Published:** 2025-08-14

**Authors:** Ha-Eun Kim, Da-Hyeon Park, Chan-Ho An, Myeong-Yoon Choi, Dongil Kim, Youn-Sik Hong

**Affiliations:** 1 Department of Computer Science and Engineering, Incheon National University, Incheon 22012, Republic of Korea; kihaeu02@inu.ac.kr (H.-E.K.); qkrek921@inu.ac.kr (D.-H.P.); list1002@inu.ac.kr (C.-H.A.); thskan123439@inu.ac.kr (M.-Y.C.); 2 Department of Physics, Incheon National University, Incheon 22012, Republic of Korea; 201800294@inu.ac.kr

**Keywords:** distracted walking detection, gait recognition, personalized machine learning, real-time activity monitoring, smartphone sensors

## Abstract

This study introduces GaitX, a real-time pedestrian behavior recognition system that leverages only the built-in sensors of a smartphone eliminating the need for external hardware. The system is capable of detecting abnormal walking behavior, such as using a smartphone while walking, regardless of whether the device is handheld or pocketed. GaitX applies multivariate time-series features derived from accelerometer data, using ensemble machine learning models like XGBoost and Random Forest for classification. Experimental validation across 21 subjects demonstrated an average classification accuracy of 92.3%, with notably high precision (97.1%) in identifying distracted walking. In addition to real-time detection, the system explores the link between gait variability and psychological traits by integrating MBTI personality profiling, revealing the potential for emotion-aware mobility analytics. Our findings offer a scalable, cost-effective solution for mobile safety applications and personalized health monitoring.

## 1. Introduction

With the widespread adoption of smartphones, using a smartphone while walking, often referred to as “smombie” behavior (a portmanteau of “smartphone” and “zombie”), emerged as a serious social concern. This behavior reduces pedestrian attention and has become a significant cause of safety-related incidents. In South Korea, 69.0% of Seoul residents reportedly use their smartphones while walking, with the highest usage rate of 86.8% among individuals in their 30s [1]. Furthermore, accidents caused by smartphone use while walking have increased 1.6 times over the past five years, with 77.0% of such incidents occurring in people under the age of 40 [2].

This issue is not unique to Korea but has gained global attention. According to the U.S. Consumer Product Safety Commission, an estimated 4600 emergency room-treated injuries occurred nationwide over the two-year period from 2019 to 2020 due to walking while distracted on the phone [3]. The rapid increase in pedestrian accidents since the proliferation of smartphones in 2009 led to 7522 traffic-related pedestrian fatalities in 2022 alone [4]. In Australia, studies have shown that approximately 20.0% of pedestrians at urban intersections were distracted due to smartphone use, significantly increasing the likelihood of dangerous encounters with vehicles [5,6]. Globally, more than 40.0% of pedestrians are estimated to use their phones while walking, and related accidents are steadily increasing regardless of age, location, or environment.

The growing demand for objective gait analysis has driven the development of sensor-based approaches capable of providing quantitative measurements beyond traditional observational methods. Inertial Measurement Unit (IMU) sensors—comprising accelerometers, gyroscopes, and magnetometers—offer several advantages, including portability, cost-effectiveness, and the ability to capture detailed movement patterns in real-world environments [7]. These capabilities make IMU-based systems particularly valuable for continuous monitoring, early detection of mobility impairments, and real-time feedback applications.

Numerous studies have employed wearable IMU sensors to investigate the impact of smartphone use on gait patterns, identifying differences in walking speed, stride length, and acceleration [8]. These works have shown the potential to quantitatively evaluate the effects of distraction on gait stability. However, wearable IMUs require external devices, limiting their usability and accessibility in real-world environments.

To address these limitations, recent advances in smartphone-embedded IMU sensor technology have enabled meaningful progress in mobile gait analysis. Modern smartphones incorporate accelerometers, gyroscopes, and magnetometers with sufficient accuracy and reliability for gait pattern recognition. Several studies have established the clinical validity of these built-in sensors, showing that smartphone accelerometers can perform on par with, or even surpass, external IMU devices across various activities and body placements [9]. Notably, built-in sensors achieve root mean square (RMS) errors below 0.3° under static conditions [10], and have demonstrated the ability to accurately capture spatiotemporal gait parameters such as walking speed, cadence, and stride length—even when the smartphone is carried naturally in a pocket [11].

Previous research has confirmed the reliability and repeatability of gait analysis using smartphone-embedded accelerometers [12], with consistent results in pattern recognition [13]. However, many of these studies relied on data collected from fixed phone placements (e.g., waist-mounted), and lacked support for real-time feedback or personalized modeling.

Smartphone-based approaches offer distinct advantages over wearable IMU systems. Unlike wearables, which often involve high equipment costs, dedicated lab environments, and time-consuming setup procedures [14], smartphones eliminate the need for external devices and require no calibration [11]. Moreover, while wearable sensors may face challenges in daily-life adoption due to intrusiveness and inconsistent placement [7], smartphones are naturally integrated into users’ routines without disrupting behavior [11]. These technological advancements make it feasible to conduct continuous gait monitoring in real-world conditions—without the constraints of laboratory infrastructure or wearable devices.

Other studies have also explored the potential to infer emotional states or personality traits from gait patterns, suggesting that walking behaviors may reflect inner psychological characteristics [15]. However, such studies were mostly post hoc and static in nature, and did not extend to real-time detection or active behavioral interventions for safety management.

Although existing research has demonstrated the potential of gait analysis and machine learning-based predictive modeling, systems capable of real-time detection and alerting of smartphone use while walking remain limited. Furthermore, efforts to implement personalized modeling that incorporates individual gait traits and emotional tendencies are scarce. Therefore, developing a system that uses only built-in smartphone sensors to detect abnormal gait behavior in real time and provide proactive warnings has emerged as a critical research challenge from both academic and practical perspectives. This study addresses that gap by exploring personalized analysis and emotion prediction in parallel, offering a novel contribution compared to prior work. In this context, emotion-aware does not refer to the detection of transient emotional states, but rather to the incorporation of stable psychological and behavioral traits—such as extraversion or introversion—into the personalized modeling process, as reflected in individual gait patterns.

Smartphone-carrying conditions during walking can be broadly classified based on device placement and usage behavior. Normal walking includes two main scenarios: (1) carrying the smartphone in a pocket without active use, and (2) holding the device in hand while walking naturally without intentional screen interaction. The latter, referred to as handheld gait, reflects typical daily behavior, where the user maintains a natural walking rhythm and arm swing without abnormal motion or visual engagement with the device.

In contrast, abnormal walking—or distracted walking—refers to active smartphone engagement while walking, such as screen viewing, texting, app usage, or media consumption. This behavior often leads to altered attention and modified gait due to cognitive load. Additionally, even in the absence of direct device interaction, sudden reductions in walking speed or irregular gait patterns may indicate distraction or compromised attention during locomotion.

Gait patterns are highly individualistic, varying across people even in identical contexts in terms of rhythm, pace, and stability. In this study, we explore the feasibility of personalized gait pattern analysis that accounts for these individual differences. To further examine the association between gait and psychological traits, we utilize each participant’s MBTI (Myers–Briggs Type Indicator) result as a label to assess the potential for classifying emotional tendencies (e.g., active vs. passive).

This study implements the following key features: First, we develop a system capable of distinguishing between normal and abnormal walking behaviors in real time using only built-in smartphone sensors. The system detects whether a user is interacting with their phone while walking and classifies it as abnormal walking regardless of whether the phone is in hand or in the pocket. Second, if abnormal walking continues for a predefined period, the system alerts the user via vibration or auditory signals, encouraging them to pay attention and walk safely. This real-time feedback function offers practical potential for preventing accidents caused by smartphone distraction while walking.

## 2. Built-In Smartphone Sensors and Feature Selection

Modern smartphones come equipped with a variety of sensors capable of capturing motion and location data. In this study, we considered the accelerometer, gyroscope, magnetometer, and GPS sensors for pedestrian gait analysis. Each sensor’s characteristics and limitations were evaluated to determine its suitability for real-time gait detection. As smartphone sensor chipsets vary by model and are often undisclosed by manufacturers, we based our descriptions on the typical specifications of widely used commercial IMU sensors, such as the BMI160 and MPU-9250 [16].

### 2.1. Accelerometers

**Figure 1:**
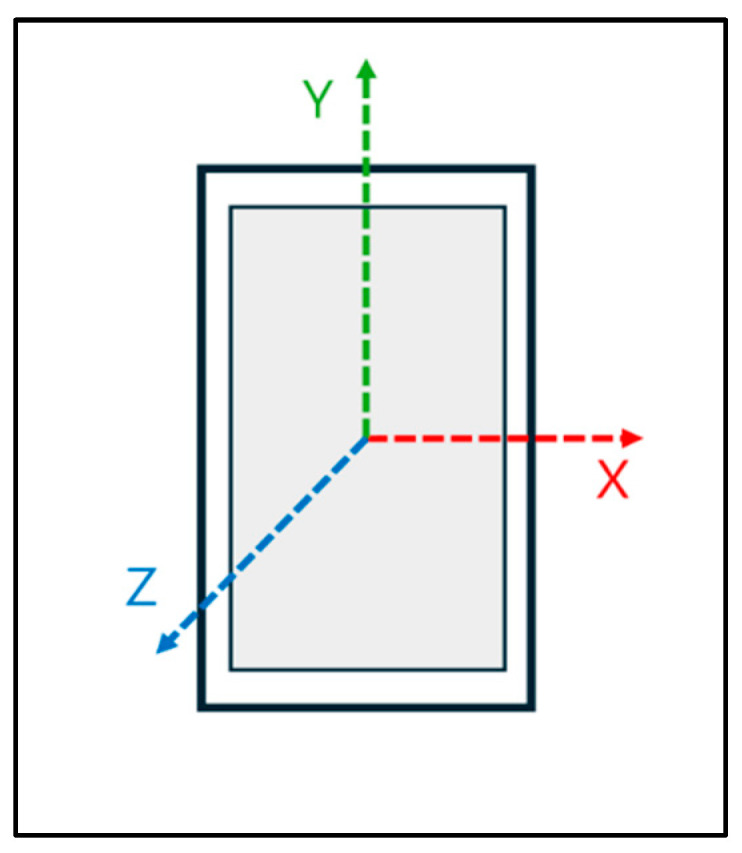
Three-axis coordinate system of a smartphone accelerometer.

The accelerometer measures linear acceleration along three orthogonal axes, as shown in Figure 1. In the smartphone’s default orientation, the X-axis corresponds to left–right movement across the screen, the Y-axis corresponds to up–down movement along the length of the screen, and the Z-axis corresponds to forward–backward movement perpendicular to the screen plane (toward or away from the user). It is essential for detecting user movement and, when combined with GPS data, can help estimate travel distance. Smartphone accelerometers typically support a measurement range of ±2 g to ±16 g, with a resolution in the range of 0.001–0.01 m/s^2^. For example, the Bosch BMI160 offers a measurement range of ±8 g with a sensitivity of 4096 LSB/g [16]. To reduce noise in the raw accelerometer data, we applied a moving average filter. Additionally, we used the Extended Kalman Filter (EKF) to compensate for the non-linear nature of gait, enabling more accurate distance estimation [17].

### 2.2. Gyroscopes

Gyroscopes measure angular velocity around the X, Y, and Z axes. Smartphone gyroscopes typically support a measurement range of ±125°/s to ±2000°/s, with a resolution of approximately 0.001–0.1°/s. For example, the Bosch BMI160 provides a sensitivity of 16.4 LSB/°/s at ±2000°/s and 262.4 LSB/°/s at ±125°/s [16]. While they can precisely track orientation changes, integrating angular velocity to estimate orientation can introduce drift error. Since we assumed straight-line walking in this study, we excluded gyroscope data from detailed analysis to avoid unnecessary rotation data.

### 2.3. Magnetometers

Magnetometers measure the Earth’s magnetic field vector across the X, Y, and Z axes. Smartphone magnetometers typically support measurement ranges from ±1300 μT to ±4800 μT, with a resolution of approximately 0.15 to 0.6 μT. However, they are highly sensitive to environmental magnetic interference, which can significantly reduce the reliability of the measurements [18]. Although they consume low power and are easy to implement, they are highly sensitive to magnetic interference, which can degrade data reliability. As this study assumed straight walking with no directional changes, magnetometer data was also excluded.

### 2.4. GPS

GPS sensors provide latitude and longitude information by receiving signals from satellites. In open environments, smartphone GPS typically exhibits an average error of approximately 3–5 m; however, in urban areas, errors can increase to 7–13 m due to signal blockage and multipath reflections caused by surrounding buildings [19]. Although they are effective for calculating movement paths and distances, they can suffer from signal interference in indoor, underground, or urban canyon environments. In particular, short-range distance estimation using only GPS is inaccurate. Thus, GPS alone is insufficient for reliable gait pattern analysis.

### 2.5. Sensor Data Acquistion and Processing for Gait Analysis 

We collected real-time data from built-in smartphone sensors, including time, acceleration (AccX, AccY, AccZ), angular velocity (GyroX, GyroY, GyroZ), magnetic field (MagX, MagY, MagZ), and GPS location (latitude, longitude), as summarized in Table 1. However, due to the limited variability in the GPS data, the latitude and longitude values were excluded from the table. Among these, acceleration data played a key role in distinguishing normal from abnormal gait, especially when analyzing periodicity, non-periodicity, and sharp changes. These findings suggest that even accelerometer data alone can effectively differentiate between regular and distracted walking behaviors.

**Figure 2:**
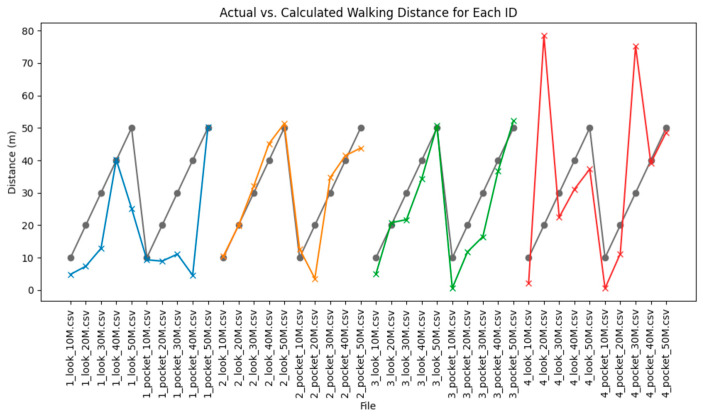
Comparison between actual (reference distance) and predicted (calculated using GPS and accelerometer data) walking distances for four participants (ID1-ID4). Different colors represent different participants.

In this study, as illustrated in Figure 2, we estimated walking distances by combining accelerometer and GPS sensor data for gait pattern analysis. Specifically, movement distances were predicted at intervals of 0.1 s and 0.01 s using accelerometer data, and accumulated errors were corrected using GPS signals. To reduce noise in the accelerometer data, a moving average filter was applied, and missing values were supplemented through interpolation [20].

The specific implementation of the sensor fusion algorithm is as follows. The state vector was defined as Equation (1) to simultaneously estimate the pedestrian’s two-dimensional position and velocity.

(1) $$X={\left[\begin{array}{c} x_{position\;}\\ x_{velocity}\\ y_{position\;}\\ y_{velocity} \end{array}\right]}^{T}$$

The state transition matrix (A) in Equation (2) was based on a constant velocity model and represents the temporal relationship between the sampling interval (Δ*t*) and velocity. The control input matrix (B) was designed to estimate changes in position and velocity using linear acceleration from the accelerometer as input.

(2) A=100dt10 00  10000dt01 B=0.5dt2dt0000.5dt2 0dt

The process noise covariance matrix (*Q*) was configured to reflect the characteristics of soft and continuous human motion, rather than abrupt changes. The sampling interval Δt used in both the state transition and control input matrices corresponds to the interpolated sensor data cycle of 0.01 s. The measurement noise covariance (*R*) was set to reflect the typical error range of GPS-based position estimation. The initial state covariance matrix ($P_{0}$) was set to enable rapid correction of uncertainty that may arise during the initial phase when aligning accelerometer data with GPS signals. The specific values used are as follows:

$$Q=0.1I_{4},\;R=5.01I_{2},\;P_{0}=300I_{4}$$

Experimental results showed that data sampled at 0.01 s intervals achieved a mean absolute error (MAE) of 9.6 m and a root mean square error (RMSE) of 15.5 m. In contrast, data sampled at 0.1 s intervals had an MAE of 13.1 m and an RMSE of 18.8 m. These results indicate that shorter sampling intervals lead to improved precision, making them more suitable for fine-grained gait analysis.

### 2.6. Sensor Selection for Gait Analysis

**Figure 3:**
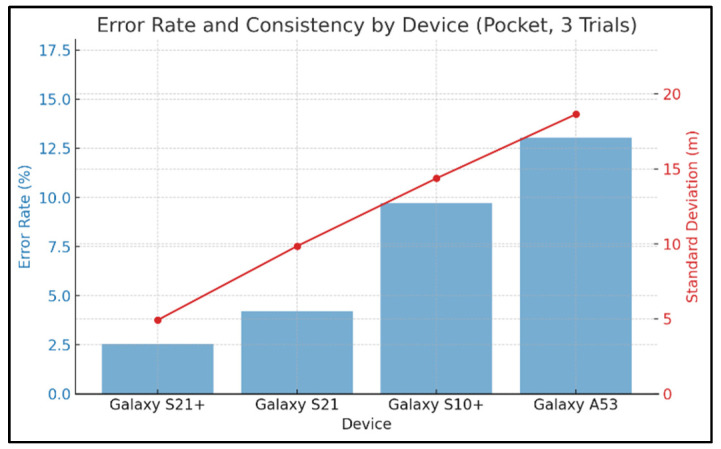
Error rates and standard deviations of predicted distances by smartphone model (pocket condition, 3 repetitions).

To examine how sensor performance affects distance measurement accuracy, we conducted distance estimation experiments using four smartphone models: Galaxy S10+ (flagship; Samsung Electronics Co., Ltd., Suwon, Republic of Korea), Galaxy S21 (flagship; Samsung Electronics Co., Ltd., Suwon, Republic of Korea), Galaxy S21+ (flagship; Samsung Electronics Co., Ltd., Suwon, Republic of Korea), and Galaxy A53 (mid-range; Samsung Electronics Co., Ltd., Suwon, Republic of Korea). Participants were instructed to walk with the smartphone placed in their pants pocket, and each trial was repeated three times for the same participant (referred to as the “pocket” condition). The error rate was calculated by comparing the predicted distance—computed from GPS and accelerometer data—to the actual walking distance. Results are presented in Figure 3.

In Figure 3, blue bars represent the error rate for each device, while the red line indicates the standard deviation. As shown, the Galaxy S21+ achieved the highest prediction accuracy (error rate: 2.55%, standard deviation: 4.92 m), followed by the Galaxy S21 (4.2%, 9.85 m), Galaxy S10+ (9.72%, 14.37 m), and Galaxy A53 (13.05%, 18.64 m).

These differences can be attributed to variations in hardware performance—particularly the GPS reception capabilities and sensor precision [21,22]. The Galaxy S21+ supports dual-frequency GPS (L1 + L5) and is equipped with a high-precision accelerometer, contributing to superior distance estimation. In contrast, the Galaxy A53 supports only single-frequency GPS (L1) and has relatively low-precision accelerometer components, resulting in higher estimation errors and greater variability.

These differences can be attributed to variations in hardware performance—particularly the GPS reception capabilities and sensor precision. The Galaxy S21+ supports dual-frequency GPS (L1 + L5) and is equipped with a high-precision accelerometer, contributing to superior distance estimation. In contrast, the Galaxy A53 supports only single-frequency GPS (L1) and has relatively low-precision accelerometer components, resulting in higher estimation errors and greater variability.

## 3. Pedestrian Pattern Analysis Using Acceleration Sensors

To identify abnormal walking patterns, it is necessary to detect irregularities in stride length or variations in walking speed compared to normal gait patterns. Unlike walking with the smartphone simply placed in a pocket, using the smartphone while walking results in minimal device movement, which leads to distinguishable differences in angular velocity measured by the gyroscope. Although our sensor analysis confirmed that accelerometer data plays a key role in gait classification, raw data selection for model training was finalized based on classification accuracy obtained from experiments with actual participants.

### 3.1. Data Acquisition

We conducted walking experiments with healthy volunteers in their twenties. Participants were instructed to walk naturally, without being consciously aware of the experiment, to ensure that their typical gait patterns were recorded. MBTI personality information was also collected with participant consent to analyze emotional traits. MBTI (Myers–Briggs Type Indicator) categorizes personalities along four dichotomies: Extraversion (E) vs. Introversion (I), Sensing (S) vs. Intuition (N), Thinking (T) vs. Feeling (F), and Judging (J) vs. Perceiving (P).

A total of 21 participants (12 males and 9 females) took part in the experiments. The smartphone models used by participants are listed in Table 2.

#### 3.1.1. Smartphone Characteristics

The GaitX application was implemented in the Android OS environment. Participants used their own smartphones (Samsung Galaxy models), with experiments conducted on four devices: Galaxy A53, S10+, S21, and S21+. All tests were performed outdoors.

#### 3.1.2. Experimental Conditions

Initial experiments involved participants walking a 50 m path once. To improve the dataset, subsequent experiments required each participant to complete five repetitions of a 100 m walk. For personalized gait analysis, each user underwent at least three measurements, each lasting 1 min and 30 s. Data collection began 10 s after pressing the start button, and the session ended with a vibration alert after 1 min and 30 s.

**Figure 4:**
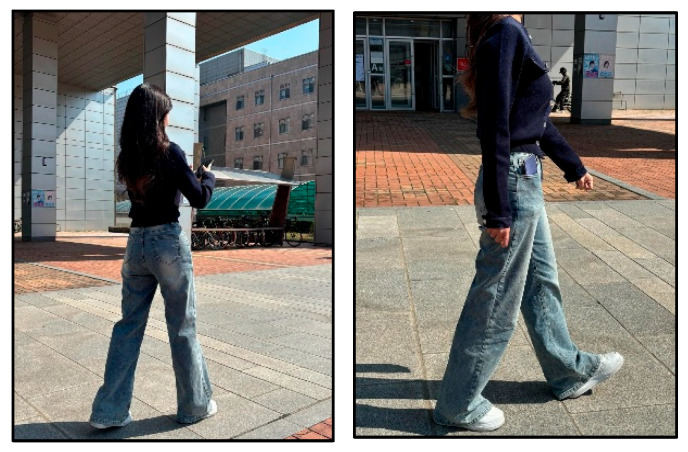
Abnormal gait (**left**) and normal gait (**right**) data collection scenarios based on smartphone usage.

In the abnormal gait conditions (referred to as “look” and “text”), participants walked while watching OTT content or sending text messages, as shown in the left image of Figure 4. In the normal gait condition (referred to as “pocket”), participants walked with the smartphone placed in their pocket without interacting with it, as shown in the right image of Figure 4.

### 3.2. Data Characteristics

The smartphone’s built-in accelerometer measures acceleration along three axes: *X*, *Y*, and *Z*. The magnitude of acceleration was calculated using Equation (3) [23]. From this, average speed, maximum speed, and rate of change were derived under different walking conditions.

(3) $$Accleration=\sqrt{{AccX}^{2}+{AccY}^{2}+{AccZ}^{2}}$$

**Figure 5:**
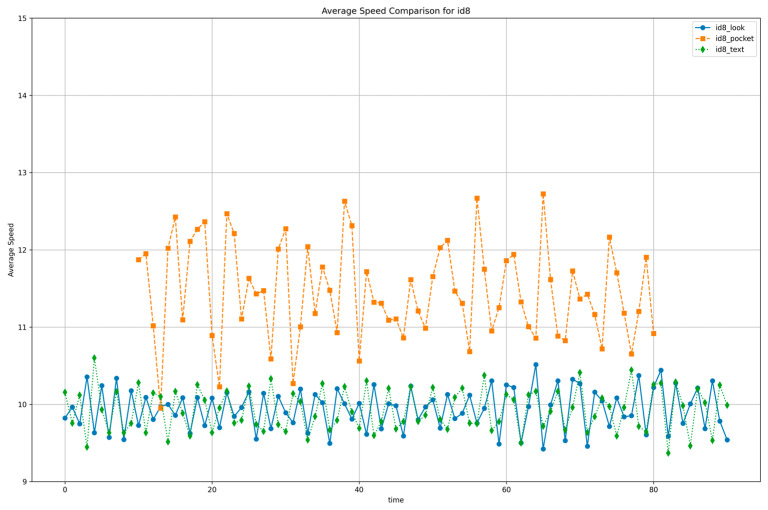
Average acceleration magnitude under three smartphone-usage conditions: pocket (orange dashed), screen-viewing (blue solid), and text messaging (green dotted).

Figure 5 illustrates that, for subject ID 8, walking with the smartphone in the pocket results in noticeably higher acceleration compared to walking while viewing the screen or sending text messages.

#### 3.2.1. Acceleration Magnitude Differences

Quantitative analysis revealed that the pocket condition yielded a mean acceleration magnitude of 12.1 ± 0.6 m·s^−2^, while the screen-viewing (“look”) and texting (“text”) conditions showed significantly lower values of 10.0 ± 0.3 m·s^−2^ and 9.9 ± 0.4 m·s^−2^, respectively. These results indicate a 17–18% reduction in mean acceleration for a representative participant (Figure 5), with the average reduction across all participants calculated at approximately 8.94%. Similarly, the standard deviation of acceleration decreased from 0.60 m·s^−2^ in the pocket condition to 0.28 m·s^−2^ and 0.27 m·s^−2^ in the screen-viewing and texting conditions, respectively, reflecting an approximate 2.2-fold reduction in gait variability.

#### 3.2.2. Acceleration Change Patterns

Under abnormal walking conditions, the overall fluctuation in acceleration was noticeably lower than during normal walking, resulting in a more consistent rhythmic pattern. Although attention is diverted while using a smartphone, users tend to unconsciously regulate their stride and maintain a steady walking pace and step length.

These findings suggest that changes in gait patterns can be meaningfully identified using only accelerometer data. In other words, the built-in accelerometer of a smartphone—without requiring any additional sensors—can effectively detect fluctuations in walking speed and movement patterns, thus enabling real-time recognition of attention-diverting behaviors.

We used features such as variability, mean, and maximum acceleration to evaluate gait. Variability reflects stability and consistency, where increased irregular motion suggests potential abnormal behavior. Mean values represent overall motion intensity, useful for detecting repetitive deviations. Maximum values capture intense or abrupt movements, which have been linked to increased fall risk [24]. Combining these three metrics enables effective differentiation between normal and abnormal walking behavior.

### 3.3. Implementation Environment

**Figure 6:**
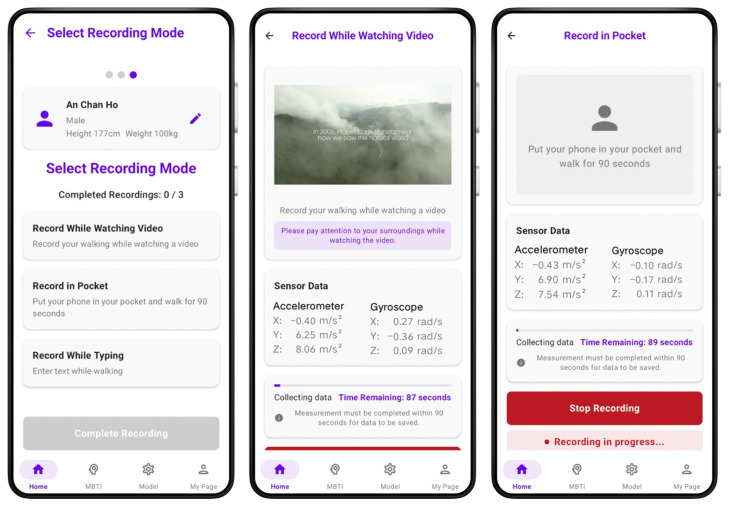
GaitX Application Screens: Menu, OTT Viewing, and Pocket Mode.

The GaitX mobile application was developed for Android OS (version 12–14). Unlike smartwatches that detect walking states automatically, GaitX requires users to activate data collection manually. Figure 6 illustrates the app’s key screens: menu selection, walking while watching OTT content, and walking with the phone in the pocket.

### 3.4. Real-Time Alerting Mechanism

The GaitX system analyzes a user’s walking data at 1 s intervals, which provides a balanced trade-off between sensor resolution, computational load, and battery consumption, while maintaining sufficient temporal resolution for real-time decision-making. If abnormal walking behavior is detected continuously over five consecutive 1 s analyses (i.e., 5 s total), a notification is triggered. The 5 s threshold was chosen to minimize false positives caused by brief screen interactions or temporary sensor noise, while still capturing genuine safety risks.

The notification is delivered as a translucent toast overlay displayed at the top of the screen, with the message: “The use of smartphones was detected during walking. Please pay attention to safety.” The message remains visible for 7 s, which was selected based on usability guidelines from the Nielsen Norman Group to ensure sufficient time for user recognition [25]. Users can manually dismiss the alert at any time by tapping the “✕” icon on the toast. To minimize user disruption, the system does not employ sound or vibration cues.

Performance tests on a prototype device running Android 12 with a Snapdragon 888 processor showed an average end-to-end delay of 120 ms (standard deviation: 25 ms), with a maximum delay of 180 ms from accelerometer sampling to toast display. These results confirm that the notification system operates well within the 1 s analysis window, meeting real-time performance requirements.

While the toast notification was successfully implemented to provide real-time alerts with minimal delay, a formal usability evaluation has not yet been conducted. This prototype implementation primarily serves to demonstrate potential real-world application and user interaction. Future research will include comprehensive usability testing to assess recognition time, response latency, user acceptance, perceived disturbance, and behavioral impact of the alerting mechanism.

## 4. Experimental Results

Based on the analyses presented in Section 3, we developed a gait pattern recognition application using smartphone accelerometer data. A personalized model was trained on individual gait patterns, enabling the classification of walking behavior into normal and abnormal categories.

### 4.1. Machine Learning Models

Gait data was collected in real time and processed at one-second intervals to calculate average speed, maximum speed, and standard deviation of speed changes. To classify gait, we applied several machine learning algorithms, including Random Forest, XGBoost, Decision Tree, K-Nearest Neighbors (KNN), Support Vector Machine (SVM), and Logistic Regression. Key hyperparameters were optimized during model training to improve classification accuracy.

Key model settings were as follows:

- Random Forest: A total of 100 trees (n_estimators = 100) were used to maximize prediction performance. The tree depth was left unrestricted to allow optimal splitting during training.
- XGBoost: Hyperparameter tuning was performed for n_estimators, max_depth, learning_rate, subsample, and colsample_bytree. The model was trained using log-loss as the evaluation metric, with random_state set to 42 to ensure reproducibility [26].
- Decision Tree: Node splitting was based on Gini impurity, and tree depth was not constrained to allow the discovery of optimal splits.
- K-Nearest Neighbors (KNN): The number of neighbors (n_neighbors) was explored within the range of 3 to 10 to identify the optimal hyperparameter. Euclidean distance was used as the metric, and equal weighting was applied to all neighbors.
- Support Vector Machine (SVM): The radial basis function (RBF) kernel was used, with the regularization parameter C explored in the range of 0.1 to 100. The gamma parameter was set to auto-scale based on the dataset characteristics.
- Logistic Regression: To prevent overfitting, L2 regularization was applied. The regularization strength parameter C was optimized in the range of 0.1 to 100. The liblinear solver was used for model training.

### 4.2. Analysis of the Machine Learning Models

We compared model performance using datasets collected from the initial (50 m × 1) and enhanced (100 m × 5) protocols. Table 3 summarizes AUC (Area Under the ROC Curve) scores for each model. Results showed that the initial dataset had low consistency and greater performance variation. For instance, Logistic Regression and SVM showed minimum AUCs of 61%, while XGBoost’s performance varied by 23% between best and worst cases.

**Figure 7:**
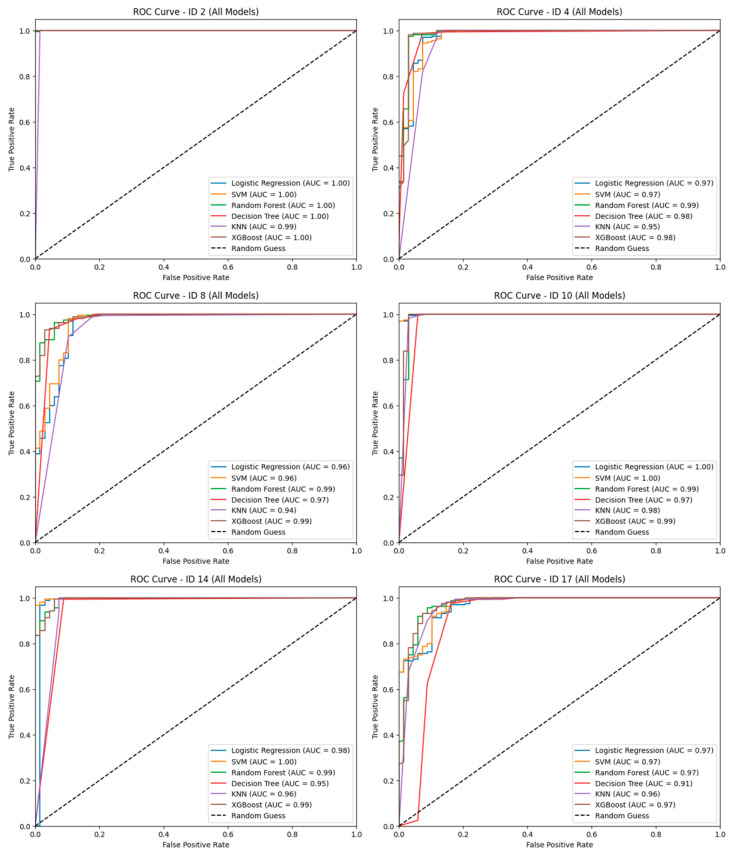
Receiver Operating Characteristics (ROC) Curves by Subject.

As summarized in Table 3, the dataset collected using the initial experimental protocol showed low AUC values due to insufficient consistency in gait patterns. Notably, there was a wide variation in performance across models. For instance, Logistic Regression and SVM exhibited a minimum AUC of 61%, while the performance range of XGBoost reached as high as 23 percentage points between its best and worst cases. In contrast, with the improved data collection protocol, the average AUC scores of all models increased, and performance variability decreased. In particular, Random Forest and XGBoost both achieved average AUCs exceeding 95%, with a maximum performance gap of less than 13%, indicating more stable results. These findings suggest that increasing the amount of data and the number of repetitions significantly contributed to enhancing the generalization capability of the models. As shown in Figure 7, ROC curves for selected subject IDs illustrate representative performance cases under the improved protocol.

- Random Forest: As an ensemble learning method that combines multiple decision trees, Random Forest demonstrated robust performance, particularly with non-linear data. One of its advantages is the ability to analyze feature importance, enabling interpretation of how specific variables influence predictions. In addition, the model exhibited relatively low variability in performance, offering consistent and practical results.
- XGBoost: XGBoost is a lightweight gradient boosting algorithm known for its high predictive accuracy and fast training speed. In this study, it consistently achieved high AUC scores across most subject IDs and showed the lowest performance variability among all models, confirming its reliability. It includes built-in mechanisms such as regularization, tree pruning, and early stopping, which help prevent overfitting, making it particularly well-suited for classifying non-linear gait data.
- Decision Tree: As a single-tree model, it is prone to overfitting depending on the dataset, resulting in greater performance variability compared to ensemble approaches.
- K-Nearest Neighbors (KNN): The computational cost of KNN increases with the size of the dataset, which can degrade its efficiency. In real-time gait analysis scenarios, where speed and responsiveness are critical, KNN may be less suitable due to these computational limitations.
- Logistic Regression and SVM: These models exhibited limited classification performance when the data was not linearly separable. This outcome suggests that gait data does not follow simple linear boundaries and often involves complex patterns.

Since gait data inherently involves continuous variations in speed, acceleration, and movement patterns, non-linear models are generally more effective than linear classifiers. This explains why ensemble models such as Random Forest and XGBoost achieved superior performance. Therefore, for classifying multivariate time-series data like gait patterns, ensemble methods were deemed the most appropriate machine learning approach.

### 4.3. Analysis of Personalized Pedestrian Pattern

**Figure 8:**
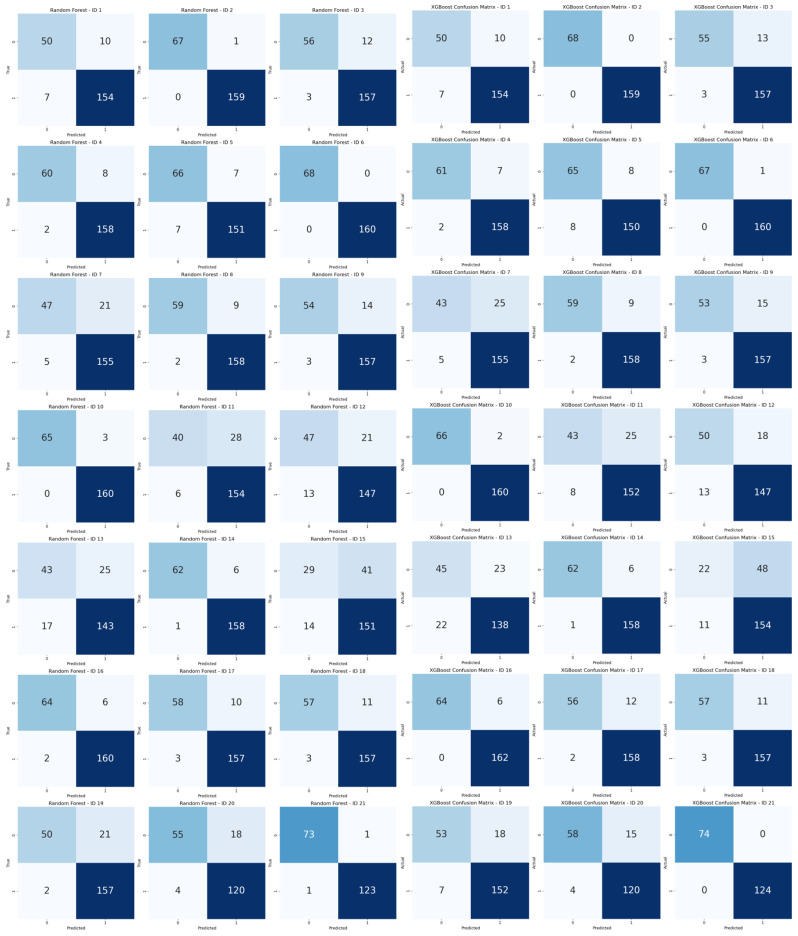
Confusion matrices for Abnormal Gait Classification (left: Random Forest, right: XGBoost).

The classification accuracy for individual gait patterns ranged from 74.9% to 100%, indicating that personalized gait characteristics can significantly influence model performance. The overall average classification accuracy was 92.2%, suggesting that while there is room for improvement in detecting abnormal walking behavior, the system achieved relatively high performance. These results are based on the performance of the Random Forest and XGBoost models, with detailed metrics presented in Table 4. A visual representation of the classification results is provided in the confusion matrix shown in Figure 8.

A more detailed performance analysis revealed that the classification accuracy for normal walking was 81.2%, while that for abnormal walking was 97.1%. This implies that the model is more effective at detecting abnormal gait patterns than normal ones, which may be attributed to the variability in individual walking characteristics.

### 4.4. Personality Types and Gait Pattern Differences

Studies utilizing wearable sensors have also reported that the four MBTI dimensions (Extraversion–Introversion, Sensing–Intuition, Thinking–Feeling, Judging–Perceiving) are significantly associated with gait characteristics. In particular, introverted participants exhibited greater changes in walking speed under conditions of visual distraction, whereas extroverted individuals showed relatively smaller variations [15].

While abnormal gait involves physically pronounced pattern changes that can be effectively classified using only accelerometer data, personality traits are based on more subtle and complex movement tendencies. Therefore, a broader range of sensor-derived features is required for more precise analysis. To enhance the detection of personality-related gait differences, we extracted additional features from various sensors. These included average and maximum speed variation (avg_speed, max_speed), standard deviation of speed change (std_speed), acceleration rate (mean_jerk, max_jerk), as well as the mean and standard deviation of gyroscope (gyro_mean, gyro_std) and magnetometer data (mag_mean, mag_std). These features reflect not only movement magnitude but also gait consistency, rotational behavior, and directional changes.

As shown in Table 5, the classification accuracy based on these multi-sensor features reached approximately 92%, representing a significant improvement over the model using accelerometer data alone. This result suggests that incorporating diverse sensor data enables the model to more effectively capture fine-grained differences in gait patterns associated with personality types. In particular, gyroscope and magnetometer-derived features contributed to the quantification of gait stability, balance maintenance, and directional shifts—characteristics that are difficult to detect using accelerometers alone—thereby enhancing overall model performance.

### 4.5. Performance Comparison Between Personalized and Generalized Models

Considering the variability in individual gait characteristics, we compared the performance of a generalized model with that of a personalized model. For the generalized model, we employed a leave-one-subject-out (LOSO) cross-validation approach, in which data from one participant were excluded during training and used solely for testing to assess cross-subject generalizability. Both models were implemented using the XGBoost algorithm to ensure a consistent basis for comparison.

As shown in Table 6, the personalized model outperformed the generalized model. For example, when data from Subject ID 16 were excluded from training and used only for evaluation, the personalized model achieved an accuracy of 80.3% and an AUC of 0.76, whereas the generalized model attained an accuracy of 75.4% and an AUC of 0.60. These results highlight the advantage of personalized modeling in capturing individual gait patterns more effectively.

## 5. Discussion

Performance analysis across different smartphone models revealed that flagship devices (e.g., Galaxy S21, S21+) achieved the highest classification accuracy (96%), followed by mid-range and older models (e.g., Galaxy A53, S10+), which still exceeded 90%. These results indicate that although hardware differences such as accelerometer resolution and noise may affect gait classification to some extent, the proposed algorithm demonstrates robust and reliable performance across general smartphone environments.

The classification accuracy for abnormal gait (97.1%) was notably higher than that for normal gait (81.2%). This asymmetry is likely attributable to the clear and consistent acceleration patterns observed during distracted walking—such as screen-viewing or texting—which are more easily distinguishable. In contrast, normal gait includes diverse natural behaviors (e.g., carrying the phone in hand or pocket), introducing greater variability and classification ambiguity.

The system was optimized for real-time analysis, using only accelerometer data to minimize processing overhead and support deployment in everyday environments. While this approach yielded over 92% accuracy in detecting abnormal gait, it was less effective for psychological profiling tasks (e.g., MBTI-based E/I classification), where accuracy improved from 68.6% using only accelerometer data to 92% when combining gyroscope and magnetometer features. This suggests that while accelerometers are well-suited for detecting distracted walking, deeper behavioral insights may require multimodal sensor fusion.

It is important to note that MBTI was used solely as an exploratory label, limited to the E/I dimension. This study does not aim to validate MBTI as a diagnostic tool, but to investigate whether self-reported behavioral tendencies correlate with observable gait differences. Acknowledging the limitations of MBTI, including its low test–retest reliability, this study treats this aspect as preliminary and hypothesis-generating.

Compared to wearable IMU-based studies reporting 95–98% accuracy, our smartphone-only method showed slightly lower performance [11,12,13]. However, it offers clear advantages in terms of cost, accessibility, and seamless integration into daily life—without requiring additional devices or calibration.

Several limitations should be noted. First, the sample predominantly comprised healthy adults in their 20s, limiting the generalizability of results across broader populations. Future studies should prioritize age-stratified validation, particularly including older adults and children, to improve external validity. Second, approximately 62% of the dataset was collected using a single device (Galaxy S10+), which may introduce device-specific bias. To address this, future work will ensure a more balanced device distribution to enable robust cross-device validation.

Additionally, data were collected on flat, unobstructed walking paths to establish a baseline for model development. Validation in more dynamic and complex environments—such as uneven terrain, stairs, and crowded public spaces—will be necessary to assess performance under real-world conditions.

## 6. Conclusions

This study presented GaitX, a real-time abnormal gait detection system leveraging only built-in smartphone sensors. By focusing on acceleration data, the system accurately identified distracted walking behaviors—such as screen-viewing and texting—while minimizing system complexity and energy consumption. The model achieved an average accuracy of 92.2%, with particularly high performance in detecting abnormal gait.

This study also explored personalized modeling by incorporating MBTI-based behavioral traits, demonstrating the feasibility of emotion-aware and user-adaptive classification. While MBTI was used solely for exploratory purposes, findings suggest the potential of integrating self-reported behavioral information into gait analytics. Furthermore, cross-device validation confirmed the robustness of the algorithm across commercially available smartphones.

Although slightly less accurate than wearable IMU-based methods, GaitX offers significant advantages in usability, cost-effectiveness, and ease of deployment, making it suitable for continuous gait monitoring in everyday life.

The current implementation also includes a real-time alert system using a toast notification designed in accordance with UI/UX usability principles. Performance testing confirmed that alerts are delivered within real-time constraints. However, formal user evaluations have not yet been conducted. To enhance practical applicability, future research will involve both qualitative and quantitative user studies to assess user experience, recognition time, notification fatigue, attention redirection, and behavioral impact.

Lastly, future work will expand data collection across diverse populations and smartphone models, validate performance in complex walking environments, and conduct long-term trials to ensure generalizability and real-world effectiveness.

## Figures and Tables

**Table 1:** Sensor Data Collected from Built-in Smartphone Modules.

Time (ms)	Acc X	Acc Y	Acc Z	Gyro X	Gyro Y	Gyro Z	Mag X	Mag Y	Mag Z
2	−10.096	−0.991	0.613	0.702	−0.318	0.027	29.58	−19.38	−34.62
25	−10.791	−0.994	0.407	0.005	−0.370	0.104	29.58	−19.38	−34.62
33	−10.518	−2.109	2.018	−0.376	−0.140	0.514	31.8	−8.28	−34.44
53	−10.697	−2.653	1.312	−0.300	−0.343	0.330	33.48	−2.7	−34.8
73	−10.367	−2.047	1.817	−0.299	−0.251	0.189	34.26	3.12	−34.32
94	−10.371	−1.633	1.590	−0.265	−0.234	0.208	34.14	3.12	−34.5

**Table 2:** Participant Profiles and Smartphone Models Used.

ID	Height (cm)	Weight (kg)	Age	Sex	MBTI	Smart Phone
1	183	85	26	male	ISTJ	Galaxy S21
2	170	70	26	male	INFJ	Galaxy S21
3	155	45	22	female	ISTP	Galaxy S10+
4	177	100	24	male	ISTP	Galaxy A53
5	162	50	24	female	ISFP	Galaxy S21+
6	176	70	26	male	ISFP	Galaxy S21+
7	160	68	22	female	INFP	Galaxy S10+
8	182	70	24	male	ESTP	Galaxy S10+
9	170	70	24	male	ESFP	Galaxy S10+
10	162	55	23	female	ENFP	Galaxy S10+
11	166	55	23	female	ESFJ	Galaxy S10+
12	167	53	22	female	ENFJ	Galaxy S10+
13	160	50	24	female	ENTJ	Galaxy S10+
14	155	65	28	female	ISTP	Galaxy S10+
15	176	70	27	male	INTJ	Galaxy S10+
16	170	75	43	male	ENTP	Galaxy S10+
17	165	60	42	female	ENFP	Galaxy S10+
18	170	70	26	male	ENTP	Galaxy S10+
19	173	70	26	male	ISFJ	Galaxy S10+
20	175	60	25	male	ISTJ	Galaxy A53
21	176	80	25	male	ENTJ	Galaxy A53

**Table 3:** Auc Performance Comparison: Initial vs. Enhanced Protocol.

	Initial Protocol			Enhanced Protocol		
	Avg.	High	Low	Avg.	High	Low
Random Forest	87.5	97	72	95.0	100	87
XGBoost	86.5	96	73	95.15	100	88
Decision Tree	78.5	91	62	91.92	100	77
KNN	83.5	97	64	91.54	99	74
SVM	81.5	95	61	92.62	100	70
Logistic Regression	81.5	95	61	93.15	100	71

**Table 4:** Protocol Classification Metrics for Abnormal Gait Detection.

Model	Accuracy	Precision	Recall	F1
XGBoost	0.9226	0.925	0.921	0.923
RandomForest	0.9243	0.927	0.923	0.924

**Table 5:** Classification Accuracy: Accelerometer-Only vs. Multi-Sensor Features for MBTI E/I.

Metric	Before Sensor Addition (Accelerometer Only)	After Sensor Addition (With Additional Sensors)
Accuracy (%)	68.59%	92.00%
Precision (%)	E: 63.01%, I: 75.58%	E: 93%, I: 92%
Recall (%)	E: 76.51%, I: 61.80%	E: 92%, I: 93%
F1—score (%)	E: 69.10%, I: 67.97%	E: 92%, I: 92%

**Table 6:** Classification Comparison of Personalized and Generalized Models (Example: Subject ID 16).

	Personalized Model	General Model
Accuracy (%)	80.3	75.4
AUC (%)	76.2	62.7
Precision (%)	85.7	77.9

## Data Availability

All data used in this paper is dependent on the sensors used and the measurement environments. The measurement values of each sensor used in our experiments will be provided upon request by e-mail.
